# Palladium(II) Complexes of NS Donor Ligands Derived from Steroidal Thiosemicarbazones as Antibacterial Agents

**DOI:** 10.3390/molecules15074784

**Published:** 2010-07-08

**Authors:** Abdullah M. Asiri, Salman A. Khan

**Affiliations:** 1 Chemistry Department, Faculty of Science, King Abdul Aziz University, P.O. Box 80203, Jeddah 21589, Saudi Arabia; 2 The Center of Excellence for Advanced Materials, King Abdul Aziz University, Jeddah 21589, P.O. Box 80203, Saudi Arabia

**Keywords:** thiosemicarbazone, palladium (II), antibacterial activity, amoxicillin

## Abstract

We have investigated the antibacterial activity of some new steroidal thiosemicarbazones and their Pd(II) metal complexes were prepared by the reaction of the thiosemicarbazones with [Pd(DMSO)_2_Cl_2_]. The steroidal thiosemicarbazones were prepared by the reaction of thiosemicarbazides with a steroidal ketone. The structures of these compounds were elucidated by IR, ^1^H-NMR, ^13^C-NMR, FAB mass spectroscopic methods, elemental analyses and TGA analysis. The antibacterial activity of these compounds were tested *in vitro* by the disk diffusion assay against two Gram-positive and two Gram-negative bacteria. The results showed that steroidal complexes are better inhibitors of both types of the bacteria (Gram-positive and Gram-negative) as compared to steroidal thiosemicarbazones. Compound **Ia** displays remarkable antibacterial activity as compared to amoxicillin.

## 1. Introduction

The chemistry of coordination metal complexes of thiosemicarbazone ligands have been receiving considerable attention primarily because of their bioinorganic relevance [1,2]. There have been attempts [3] to determine structural correlations between metal ion complexes of thiosemicarbazones and their wide spectrum of biological applications. Thiosemicarbazones have been the subject of extensive investigations. In several cases, the pharmacological action of the thiosemicarbazons is enhanced due to the presence of coordination metal ions [4,5]. It is well authenticated that a NS bidentate system is present in most of the thiosemicarbazones having carcinostatic potency and possessing substantial *in vitro* activity against various human tumour lines [6,7]. The thiosemicarbazone derivatives of Pd(II) have proven to be more effective as anticancer or anti-microbial agents than the ligand by itself , probably due to the increased lipophilicity of the complexes as compared to the free ligands alone [8]. Recent spectral and structural studies of Pd(II) complexes of *N*-substituted thiosemicarbazones showed a diversity of molecular geometries of the compounds, depending on the ligand, the salt used in synthesis and the method of compound synthesis. Substitution on the terminal N position can also affect the coordination and biological properties. In this paper, we report herein synthesis, characterization and antibacterial activity of Pd (II) complexes of steroidal thiosemicarbazones (STSC) against two Gram-positive and Gram-negative bacteria.

## 2. Results and Discussion

**Scheme 1:**
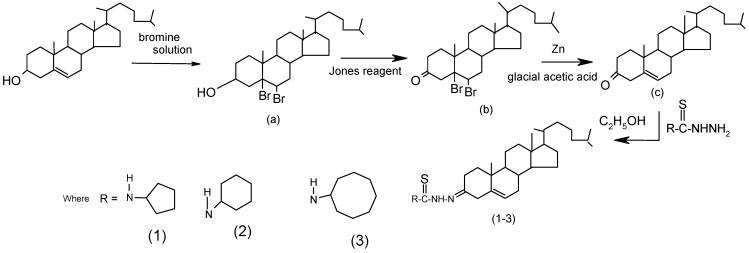
Synthesis of the steroidal thiosemicarbazones **1-3**.

**Scheme 2:**
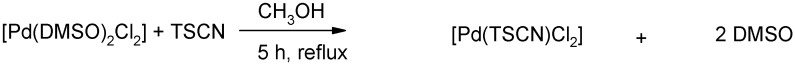
Showing the synthesis of complexes (**1a-3a**).

Reaction of steroidal thiosemicarbazones with [Pd(DMSO)Cl_2_] gave amorphous solid compounds. All the compounds were isolated in good yields and were stable both in the solid and solution state. The structures of the ligands and complexes presented in Scheme 1, Scheme 2 and Figure 2 were established by comparing spectral data (IR, ^1^H-NMR, ^13^C-NMR and FAB mass spectra) with the free ligand, along with their thermogravimetric analysis.

where TSCN = thiosemicarbazones.

### 2.1. IR spectral studies

**Figure 1:**
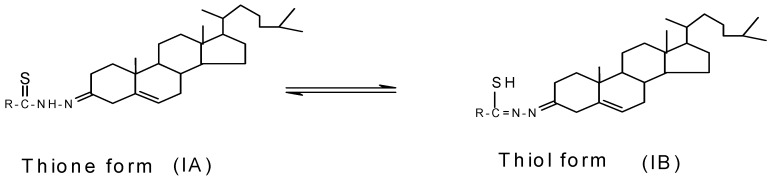
Structure of thione and thiol form of thiosemicarbazones.

Assignments of selected characteristic IR band positions provide significant indication for the formation of steroidal thiosemicarbazones and their complexes. The thiosemicarbazones can exist as thione and thiol tautomeric froms **IA** and **IB** (Figure 1), respectively. 

However, the existence of a strong band in the region 1,026–1,038 cm^-1^ due to *v* (C=S) and absence of any band in the region 2,500–2,600 cm^-1^ due to *v* (C-SH) suggested that all the thiosemicarbazones remain in their thione form. 

**Figure 2:**
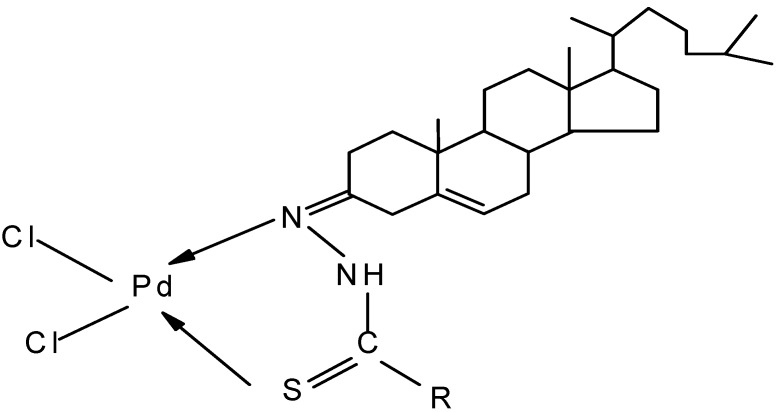
Structure of palladium (II) complexes: (**1a**) R= -NHC_5_H_9._

A strong band at 1,565-1,578 cm^-1^ was assigned to the *v* (C*=*N) stretch of the azomethine linkage in the spectra of the free ligands. In the complexes this band shifted to lower frequency by 27–38 cm^-1^ and this lowering was attributed to the coordination of azomethine nitrogen with metal and formation of M-N band. The strong band at 1,018–1,024 cm^-1^ ascribed to *v* (C=S) of ligands is shifted to lower frequency (by 8–14 cm^-1^), indicating the bonding of metal through thionic sulphur. This contention was further confirmed by the presence of *v* (Pd-N) and *v* (Pd-S) bands at 508–521 and 428–437 cm^-1^ in the far IR region of the complexes. The broad band observed in region 3,242–3,256 cm^-1^ due to *v* (N-H) stretch is only slightly affected by the coordination.

### 2.2. NMR spectral analysis

The structures of thiosemicarbazones and their metal complexes were further confirmed from the ^1^H-NMR spectra, which provide diagnostic tools for the positional elucidation of the protons. Assignments of the signals are based on the chemical shifts and intensity patterns. The ^1^H-NMR spectra of thiosemicarbazones **1-3** recorded in DMSO-d_6_ exhibit a broad peak at 9.84–10.48 ppm due to -NH proton, which indicate that even in a polar solvent they remain in the thione form. The -NH proton signal of the thiosemicarbazones usually shifts to upfield and appears at 3.38–3.82 ppm in their respective complexes. This information suggests the adjustment of electronic current upon coordination of C=S group to the metal ion. 

The ^13^C-NMR spectra of ligands were recorded in DMSO-d_6_ and the spectral signals were in good agreement with the proposed structures. The ligands showed signals at 185.8–186.6 ppm due to (C=S) and 153.8–155.7 ppm assigned to (C=N), respectively complex 182.2–184.5 ppm and 152.2–156.7 ppm assigned to (C=S) and (C=N) respectively. Other carbons in this complex resonate nearly at the same frequency as that of free ligands as given in the Experimental.

### 2.3. Thermogravimetric analysis

The TGA profiles of complexes (under nitrogen, 10% min) along with the % weight at different temperatures were recorded. These complexes do not lose weight up to 240 ºC. Further increment of temperature causes decomposition of the complexes in two steps. The temperature range for the first step was 240–312 ºC. In complexes **1a**, **2a** and **3a**, the first fragment corresponded to the loss of chlorine and sulfur atoms from complexes. The second step starts immediately after the first step and continues until the complete decomposition of the ligands and formation of the end product as palladium sulfide (PdS). The total % weight loss of the complexes corresponds to the loss of respective ligands after considering the transfer of one sulfur atom to the metal ion and the residues correspond to the palladium sulfide.

### 2.4. FAB mass analysis

Characteristic peaks were observed in the mass spectra of ligands and their metal complexes, which followed the similar fragmentation pattern. The spectrum of compound **1** showed a molecular ion peak (M^+.^) at m/z 526 and its complex compound **1a** showed a molecular ion peak (M^+.^) at m/z 701. The characteristic peaks observed within the mass spectra of thiosemicarbazones and their metal complexes are given in the Experimental section.

### 2.5. In-vitro anti-bacterial activity

The *in vitro* antibacterial activity of steroidal thiosemicarbazones and their metal complexes were assayed by the disk diffusion method using cultures of *S. aureus, S. pyogenes, S. typhimurium*, and *E. coli* [12]. Amoxicillin (30 mg) was used as the standard drug, whereas a DMSO-wetted disk was used as negative control. Results showed that metal complexes are better antibacterial agent as compared to the parent thiosemicarbazones. Results are summarized in Table 1.

**Table 1:** Antibacterial activity of steroidal thiosemicarbazones and their complexes, Positive control **A** (Amoxicillin), and negative control (DMSO). Measured by the Halo Zone Test (unit, mm).

Compound	Corresponding effect on microorganisms			
	*S. aureus*	*S. Pyogenes*	*S. typhimurium*	*E. coli*
**1**	15.2 ± 0.4	16.8 ± 0.4	16.4 ± 0.2	14.2 ± 0.5
**2**	13.4 ± 0.4	12.2 ± 0.5	11.6 ± 0.4	13.2 ± 0.5
**3**	11.2 ± 0.4	13.5 ± 0.3	10.9 ± 0.5	12.2 ± 0.2
**1a**	21.5 ± 0.5	23.2± 0.4	25.6 ± 0.2	21.6 ± 0.4
**2a**	16.6 ± 0.4	15.4 ± 0.2	16.2 ± 0.2	15.8 ± 0.4
**3a**	15.5 ± 0.3	16.7± 0.4	14.5 ± 0.5	16.8 ± 0.5
A.	21.0 ± 0.5	22.2 ± 0.4	25.2 ± 0.8	20.0 ± 0.2
DMSO	-	-	-	-

## 3. Experimental

### 3.1. General

All melting points were measured with a capillary apparatus and are uncorrected. All the compounds were routinely checked by IR, ^1^H-NMR, ^13^C-NMR, mass spectrometry and elemental analysis. IR spectra were recorded in KBr on a Perkin-Elmer model 1620 FTIR spectrophotometer. ^1^H- NMR and ^13^C-NMR spectra were recorded at ambient temperature using a Brucker Spectroscopin DPX-600 MHz spectrophotometer in CDCl_3_ and DMSO. The following abbreviations were used to indicate the peak multiplicity s- singlet, d- doublet, t- triplet, m- multiplet. FAB mass spectra were recorded on a JEOL SX102 mass spectrometer using Argon/Xenon (6 kV, 10 mB gas). Column chromatography was performed on silica gel (Merck). The reactions were monitored by precoated aluminium silica gel 60F 254 thin layer plates procured from Merck (Germany). Anhydrous sodium sulfate was used as a drying agent for the organic phase. Compounds a, b, c and thiosemicarbazide were prepared according to published methods [9]. 

### 3.2. Synthesis of thiosemicarbazones: A general method

Steroidal thiosemicarbazones were synthesized (Scheme 1) by refluxing a solution of thiosemicarbazide (0.03 mol) in methanol (15 mL) and the alcoholic solution of steroidal ketones (0.03 mol, 11.52 g, 15 mL) at 60 ºC for 5 h with continuous stirring. After cooling the compounds were filtered and recrystallized from methanol [10]. 

*Cholest-5-en-3-one cyclopentyl thiosemicarbazone* (**1**). C_33_H_55_N_3_S;Yield: 68.5%; m.p. 206–208 ºC; IR (KBr) ν *_wax_* cm^-1^: 3242 (N-H), 1565 (C=N), 1622 (C=C), 1126 (C-N), 1038 (C=S); ^1^H-NMR (DMSO-d_6_) (*δ*, ppm): 10.45 (2H, s, -NH), 4.16 (m, 8H, -CH_2_), 5.34 (1H, s, C6-H), 1.08 (s, C10-CH_3_), 0.78 (s, C13-CH_3_), 0.84, 0.96 (other methyl protons); ^13^C-NMR (DMSO-d_6_) (*δ,* ppm): 186.6 (C=S), 155.7 (C=N), 134.8 (C-NH), 22.6 (C10-CH_3_), 19.8 (C13-CH_3_); Mass spectra (M^+.^) at m/z 526, 457 (M-C_5_H_9_), 542 (M-C_5_H_10_N), 398 (M- C_6_H_10_NS), 383 (M- C_6_H_11_NS); Anal. Calc. for C_33_H_55_N_3_S: C, 75.42; H, 10.47; N, 8.00. Found: C, 75.28; H, 10.32: N, 7.93.

*Cholest-5-en-3-one cyclohexyl thiosemicarbazone* (**2**). C_34_H_57_N_3_S; Yield: 72.00%; m.p. 216–218 ºC; IR (KBr) ν *_wax_* cm^-1^: 3252 (N-H), 1572 (C=N), 1628 (C=C), 1132 (C-N), 1034 (C=S); ^1^H-NMR (DMSO-d_6_) (*δ*, ppm): 9.84 (2H, s, -NH), 4.12 (m, 10H, -CH_2_), 5.38 (1H, s, C6-H), 1.12 (C10-CH_3_), 0.82 (C13-CH_3_), 0.88, 1.02 (other methyl protons); ^13^C-NMR (DMSO-d_6_) (*δ*, ppm): 185.2 (C=S), 154.5 (C=N), 132.5 (C-NH), 23.5 (C10-CH_3_), 18.9 (C13-CH_3_); Mass spectra (M^+.^) at m/z 540, 457(M-C_6_H_11_), 542 (M-C_6_H_12_N), 398 (M- C_7_H_12_NS), 383 (M- C_7_H_13_N_2_S); Anal. Calc. for C_34_H_57_N_3_S: C, 75.69; H, 10.57; N, 7.79. Found: C, 75.62; H, 10.49: N, 7.75.

*Cholest-5-en-3-one cyclooctyl thiosemicarbazone* (**3**). C_36_H_61_N_3_S; Yield: 75.5%; m.p. 228–230 ºC; IR (KBr) ν *_wax_* cm^-1^: 3256 (N-H), 1578 (C=N), 1632 (C=C), 1138 (C-N), 1026 (C=S); ^1^H-NMR (DMSO-d_6_) (*δ,* ppm): 10.48 (2H, s, -NH), 4.22 (m, 14H, -CH_2_), 5.32 (1H, s, C6-H), 1.10 (C10-CH_3_), 0.80 (C13-CH_3_), 0.90, 1.04 (other methyl protons); ^13^C-NMR (DMSO-d_6_) (*δ*, ppm): 185.8 (C=S), 153.8 (C=N), 134.8 (C-NH), 24.5 (C10-CH_3_), 20.2 (C13-CH_3_); Mass spectra (M^+.^) at m/z 568, 459 (M-C_8_H_13_), 444 (M-C_8_H_14_N), 400 (M- C_9_H_14_NS), 385 (M- C_9_H_15_N_2_S); Anal. Calc. for C_36_H_61_N_3_S: C, 76.19; H, 10.75; N, 7.40, Found: C, 76.12; H, 10.68: N, 7.35.

### 3.3. Preparation of palladium (II) complexes

The metal complexes were prepared by mixing an equimolar ratio of ligand and [Pd (DMSO)_2 _Cl_2_] in refluxing methanol. The solution was kept at 0 ºC overnight, the product was separated by filtration and finally washed with methanol. Recrystallization was effected in methanol/ DMF (6:4) [11].

*Dichloro(cholest-5-en-3-one cyclopentyl thiosemicarbazone)palladium (II)* (**1a**). Pd (C_33_H_55_N_3_S) Cl_2_); Yield: 76%; m.p. 256–258 ºC; IR (KBr) *v _wax_* cm^-1^: 3442 (N-H), 1538 (C=N), 1528 (C=C), 1152 (C-N), 1024 (C=S), 516, 434 (Pd-N, Pd-S); ^1^H-NMR (DMSO-d_6_) (*δ*, ppm): 9.54 (2H, s, -NH), 5.58 (1H, s, C6-H), 4.22 (m, 8H, -CH_2_), 3.82 (1H, s, NH), 1.12, (C10-CH_3_), 0.82 (C13-CH_3_), 0.94, 1.06 (other methyl protons); ^13^C-NMR (DMSO-d_6_) (*δ*, ppm): 184.5 (C=S), 153.8 (C=N), 133.8 (C-NH), 22.2 (C10-CH_3_), 20.4 (C13-CH_3_), 19.4, 19.8 (other methyl carbon); Mass spectra (M^+^.) at m/z 701, 666 (M- Cl), 731 (M-Cl_2_), 597 (M-Pd), 632 (M-C_5_H_9_), 617(M- C_5_H_10_N), 573 (M- C_6_H_10_NS), 558 (M- C_6_H_11_N_2_S). Anal. Calc. for Pd(C_33_H_55_N_3_S)Cl_2_: C, 56.38; H, 7.83; N, 5.98, Cl, 10.09, Pd, 15.12. Found: C, 56.34; H, 7.79; N, 5.95, Cl, 10.02, Pd, 15.09.

*Dichloro(cholest-5-en-3-one cyclohexyl thiosemicarbazone)palladium (II)* (**2a**). Pd (C_35_H_57_N_3_S) Cl_2_; Yield: 72.00%; m.p. 264–265 ºC; IR (KBr) ν *_wax_* cm^-1^: 3456 (N-H), 1545 (C=N), 1538 (C=C), 1156 (C-N), 1022 (C=S), 508, 428 (Pd-N, Pd-S); ^1^H-NMR (DMSO-d_6_) (*δ*, ppm): 9.56 (2H, s, -NH), 4.16 (m, 10H, -CH_2_), 5.52 (1H, s, C6-H), 3.38 (1H, s, NH), 1.16 (C10-CH_3_), 0.86 (C13-CH_3_), 0.92, 1.06 (other methyl protons); ^13^C-NMR (DMSO-d_6_) (*δ,* ppm): 183.5 (C=S), 152.2 (C=N), 131.5 (C-NH), 22.4(C10-CH_3_), 18.4 (C13-CH_3_); Mass spectra (M^+^.) at m/z 715, 680 (M- Cl), 645 (M-Cl_2_), 611 (M-Pd) 632 (M-C_6_H_11_), 617 (M-C_6_H_12_N), 573 (M- C_7_H_12_NS), 558 (M- C_7_H_13_N_2_S); Anal. Calc. for Pd (C_34_H_57_N_3_S) Cl_2_: C, 56.95; H, 7.98; N, 5.86, Cl, 9.89, Pd, 14.85. Found: C, 56.89; H, 7.95; N, 5.85, Cl, 9.85, Pd, 14.82.

*Dichloro(cholest-5-en-3-one cyclooctyl thiosemicarbazone)palladium (II)* (**3a**). Pd(C_36_H_61_N_3_S)Cl_2;_Yield: 76.8%; m.p. 272–273 ºC; IR (KBr) ν *_wax_* cm^-1^: 3438 (N-H), 1532 (C=N), 1522(C=C), 1172 (C-N), 1018 (C=S), 521, 437 (Pd-N, Pd-S); ^1^H-NMR (DMSO-d_6_) (*δ*, ppm): 9.45 (2H, s, -NH), 4.12 (m, 14H, -CH_2_), 5.62 (1H, s, C6-H), 3.65 (1H, s, NH), 1.14 (C10-CH_3_), 0.84 (C13-CH_3_), 0.96, 1.04 (other methyl protons); ^13^C-NMR (DMSO-d_6_) (*δ*, ppm): 182.2 (C=S), 156.7 (C=N), 128.4 (C-NH), 23.4 (C10-CH_3_), 19.5 (C13-CH_3_); Mass spectra (M^+.^) at m/z 742, 707 (M-Cl), 672 (M-Cl_2_), 637 (M-Pd), 633 (C_8_H_13_), 618 (M-C_8_H_14_N), 574 (M-C_9_H_14_NS), 559 (M-C_9_H_15_N_2_S); Anal. Calc. for Pd(C_36_H_61_N_3_S)Cl_2_: C, 58.03; H, 8.19; N, 5.64, Cl, 9.52, Pd, 14.29. Found: C, 58.01; H, 8.18: N, 5.62, Cl, 9.48, 14.26.

### 3.4. Organism culture and in vitro screening

Antibacterial activity was assayed by the disk diffusion method with minor modifications. *S. aureus, S. pyogenes, S. typhimurium,* and *E. coli* were subcultured in BHI medium and incubated for 18 h at 37 ºC, and then the bacterial cells were suspended, according to the McFarland protocol in saline solution to produce a suspension of about 10^-5 ^CFU mL^-1^. Ten μL of this suspension was mixed with sterile antibiotic agar (10 mL) at 40 ºC and poured onto an agar plate in a laminar flow cabinet. Five paper disks (6.0 mm diameter) were fixed onto nutrient agar plate. Ten mg of each test compound was dissolved in DMSO (100 μL) to prepare stock solution and from stock solution different concentration of 10 (1 μL stock solution + 9 μL solvent), 20 (1 μL stock solution + 4 μL solvent), 25 (1 μL stock solution + 3 μL solvent), 50 (1 μL stock solution + 1 μL solvent), and 100 μg/μL of each test compound were prepared. These compounds of different concentration were poured over disk plate on to it. Amoxicillin (30 μg) was used as standard drug (positive control). A DMSO-wetted disk was used as negative control. The susceptibility of the bacteria to the test compounds was determined by the formation of an inhibitory zone after 18 h of incubation at 36 ºC. Table 1 reports the inhibition zones (mm) of each compound and the controls this experiment was repeated two times for each compound and found same results.

## 4. Conclusions

This research examined the synthesis, characterization and antibacterial activity of some new steroidal thiosemicarbazone derivatives and their Pd (II) complexes. *In vitro* antibacterial activity of these compounds was tested by the disk diffusion assay against two gram-positive and gram-negative bacteria. The result showed that cyclopentyl steroidal thiosemicarbazone derivative was found the most active among all thiosemicarbazones tested. Compound Ia displays remarkable antibacterial activity as compared to amoxicillin.
